# Case Report: Prenatal Diagnosis for a Rett Syndrome Family Caused by a Novel *MECP2* Deletion With Heteroduplexes of PCR Product

**DOI:** 10.3389/fped.2021.748641

**Published:** 2021-10-27

**Authors:** Honghong Zhang, Yixi Sun, Yuxia Zhu, Jiali Hong, Miaomiao Zheng

**Affiliations:** ^1^Department of Pediatrics, Hangzhou Children's Hospital, Hangzhou, China; ^2^Department of Reproductive Genetics, Women's Hospital, School of Medicine, Zhejiang University, Hangzhou, China; ^3^Key Laboratory of Reproductive Genetics, Ministry of Education, Zhejiang University, Hangzhou, China

**Keywords:** Rett syndrome, *MECP2*, prenatal diagnosis, mosaicism, heteroduplexes

## Abstract

Rett syndrome is an X-linked dominant, postnatal neurological disorder. Approximately 80–90% of classic Rett syndrome patients harbor mutations in the coding region of *MECP2*. Somatic or germline *MECP2* mosaicism is not rare, and paternal germline *MECP2* mosaicism occurs in especially high proportions. Here, we report the case of a Chinese girl with Rett syndrome in whom a heterozygous deletion was found in exon 4 of *MECP2* using multiplex ligation-dependent probe amplification. To obtain an accurate region of deletion, we narrowed down the deletion region using real-time quantitative PCR, and subsequent long-range PCR was performed to detect the deletion breakpoints. Surprisingly, three DNA bands from long-range PCR products were observed after gel electrophoresis. To exclude somatic mosaicism, we performed T-A cloning and DNA sequencing, the middle DNA band was proved to be a heteroduplex of the PCR product *in vitro*. Meanwhile, a prenatal diagnosis was performed for the pregnant mother of the patient. Our study showed that the patient was heterozygous for the deletion of 713-base pairs in exon 4 of *MECP2* (*MECP2*: c.441_1153del713), resulting in a frameshift and premature termination of the 487 amino acid protein at the 154th codon. In summary, we reported a novel heterozygous deletion in the *MECP2* gene with heteroduplexes of the PCR product *in vitro*, which can help in the genetic counseling and prenatal diagnosis of disorders of *MECP2* defects.

## Introduction

Rett syndrome (MIM#312750) is an X-linked, dominant, post-natal neurological disorder with a prevalence of ~1 in 10,000–15,000 female births. Patients with classic Rett syndrome display delayed development between the period of 6–18 months, after a period of apparently normal development. It is characterized by a distinctive set of clinical features, including loss of motor skills and communicative abilities, acquired microcephaly, and the development of stereotypical hand movements (1).

In 1999, mutations in the X-linked gene, methyl-CpG-binding protein 2 (*MECP2)* at Xq28 were first reported in patients with Rett syndrome. Approximately 80–90% of classic Rett syndrome patients harbor mutations in the coding region of *MECP2*, and approximately up to 15% of these mutations are gross deletions and other rearrangements, which can be detected by MLPA or southern blot (2–7).

*MECP2* mutations have been reported in patients with somatic mosaicism; females or even males with somatic *MECP2* mosaicism can survive to birth and exhibit clinical features similar to a full mutation in females or an atypical Rett syndrome phenotype (8–11). Paternal or maternal germline *MECP2* mosaicism are also not rare (2, 12–14). Therefore, special attention should be paid to somatic and germline *MECP2* mosaicism. In this study, we present a novel heterozygous deletion in the *MECP2* gene in a Chinese girl with Rett syndrome. A variety of methods (including multiplex ligation-dependent probe amplification (MLPA), real-time quantitative PCR (qPCR), long-chain PCR, DNA sequencing, gel electrophoresis, and T-A cloning) were used to detect the precise mutation, excluding somatic mosaicism. We also performed prenatal diagnosis for her pregnant mother to avoid germline *MECP2* mosaicism.

## Case Presentation

The pedigree is a family from Zhejiang province in China. The proband was a 4-year-old girl who was diagnosed with typical Rett syndrome. This girl was born by normal delivery at 38 weeks gestation. She held up her head at 3 months and could sit at 6 months, and she was able to stand with support at 10 months. At 11 months she had said “papa” and “mama,” but after 13 months she no longer spoke these words. Emotionally she was noted to be more distant and no longer responded to her parents' call by turning her head toward them. Subsequent loss of acquired purposeful hand skills began at 15 months, and has since developed a range of stereotypical hand movements such as frequent hand wringing. Epilepsy had previously developed at 30 months and was managed with sodium valproate and lamotrigine. However, her molecular diagnosis was unclear, and her healthy mother was 20 weeks along in her pregnancy. Considering the risk of recurrence, the affected pregnant woman requested prenatal diagnosis for her fetus who may have also been at risk of Rett syndrome. This study was approved by the Ethics Committee of Women's Hospital affiliated to the Zhejiang University School of Medicine, and informed consent was obtained from all subjects.

To perform the prenatal diagnosis, we first had to know the genetic basis of Rett syndrome in the proband. Peripheral blood samples were drawn from three family members, and genomic DNA was extracted using standard protocols. We carried out a routine polymerase chain reaction (PCR) to amplify the encoding exons of *MECP2*; exon 4 (exon 3 before the discovery of a new 5 UTR exon) was amplified as five overlapping fragments (4a, 4b, 4c, 4d, and 4e) (12), and no mutations were found in the four exons of *MECP2*.

**Figure 1:**
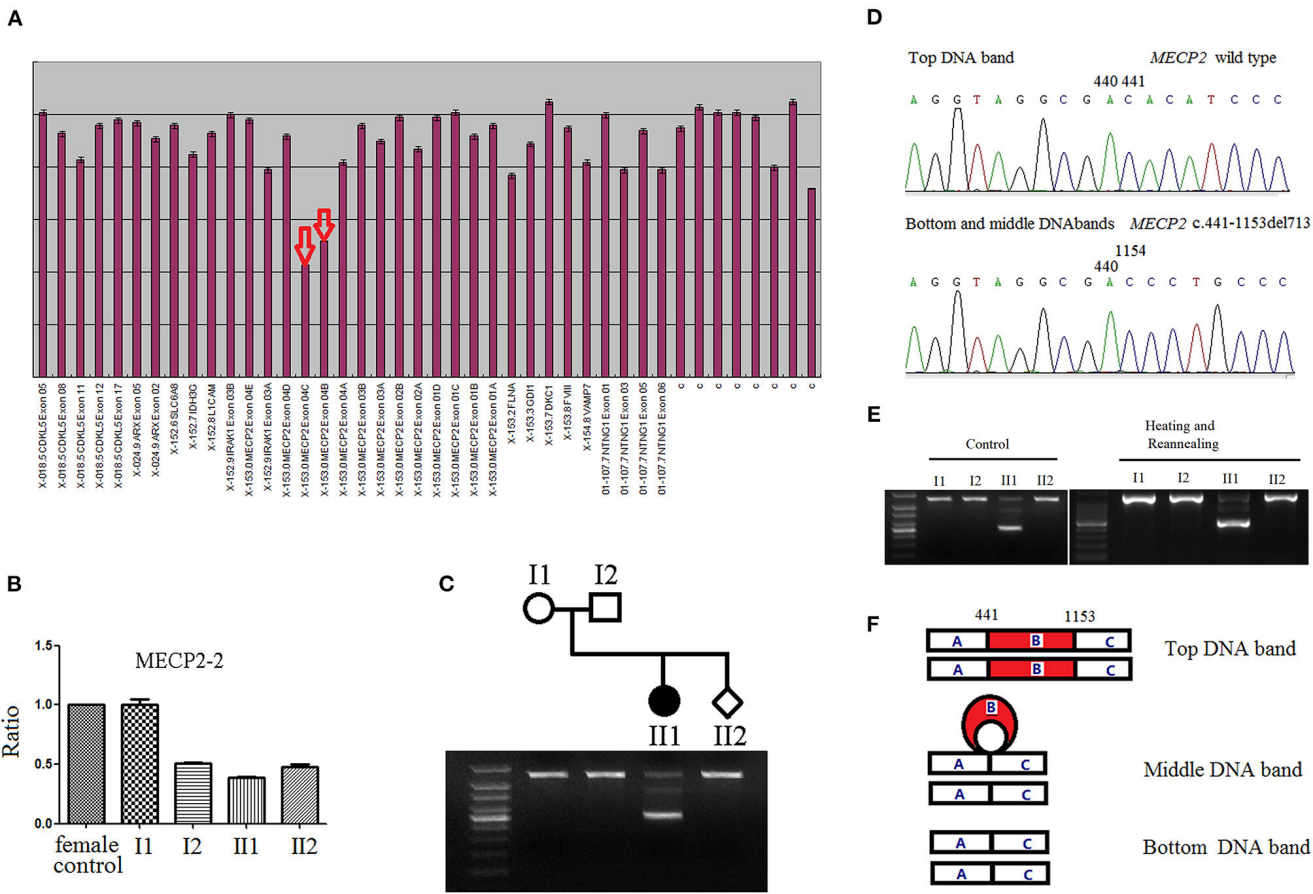
**(A)** Detection of *MECP2* exon alterations via multiplex ligation-dependent probe amplification (MLPA). The arrows illustrate the presumed deletions, as the area of the peaks from the female proband DNA is approximately half that of the female controls. **(B)** The *MECP2* amplicon with *MECP2*-real time quantitative PCR-2 primers to narrow down the deletion breakpoints and determine the results of MLPA. *MECP2* relative amount of female proband, and male fetus were half that of female control. **(C)** The products of long-range PCR were analyzed with 1.5% agarose gel electrophoresis. The PCR product from the proband showed three DNA bands, whereas PCR product bands of the proband's parents and the fetus, were single. **(D)** Three DNA bands of long-range PCR from the proband were separately cut, T-A cloned, and finally sequenced after plasmid extraction. The sequence of top DNA band was wild type; both, bottom and middle DNA bands show 713-base pairs deletion in exon 4 (c.441_1153del713), and the single band of proband's parents and the fetus were of the wild type. **(E)** There was no difference between the PCR products untreated (left) and treated with heating and reannealing (right) in electrophoresis analysis. The treated PCR products were denatured at 95 °C for 3 min, followed by gradual reannealing for 30 min with a temperature ramp of −1 °C/min to optimize for the formation of heteroduplexes and homoduplexes. **(F)** Schematic diagram of three DNA bands after PCR. The top DNA band was homoduplex of wild type, the bottom DNA band was homoduplex of mutant type, the middle DNA band was heteroduplex of wild type and mutant type. Part B (red): 713-base pairs deletion in exon 4 (c.441_1153del713) of MECP2.

We then detected the deletions using MLPA (MRC-Holland, kit P015C), which covers all four *MECP2* exons and the flanking genes *IRAK1, L1CAM*, and *SYBL1*. MLPA screening suggested that the proband had a hemizygous copy number for MLPA probes 10842-L12494 and 01347-L12498. Additionally, a heterozygous deletion of exon 4 in *MECP2* was found in the proband (Figure 1A).

We then used three pairs of real-time qPCR primers to narrow down and identify the deletion breakpoints (see Supplemental Information, Supplementary Figures 1A,B, Figure 1B). The product of long-range PCR (upon running a gel electrophoresis) showed three DNA bands of the proband (Figure 1C). The DNA was cut and purified using a DNA gel extraction kit, and the DNA bands were cloned into the pMD19-T vector via T/A ligation. After plasmid extraction, a PCR and a DNA sequencing were performed. The DNA sequence of the top band was the same as the wild-type. Surprisingly, not only the bottom band, but also the middle one, showed a 713-base pair deletion in exon 4 of *MECP2* (c.441_1153del713) (Figure 1D).

We then identified that the middle band was a heteroduplex of the wild chain and a c.441_1153del713 mutated chain. As shown in Figure 1F, for base pairing, the mutated chain combined with the wild chain around the common parts (parts A and C), but the unpaired part (part B) of the wild chain may form a particular structure. PCR amplification of the wild chain cannot be performed; therefore, the PCR product of the middle band only showed the c.441_1153del713 mutation of *MECP2*. As shown in Figure 1E, by the classical method of producing heteroduplexes, untreated PCR products (left) and those treated with heating and re-annealing were the same, further proving that the middle band was heteroduplex (Figure 1E).

In summary, we found a novel heterozygous deletion in c.441_1153del713 of *MECP2* (NM_004992.4) in the proband. This mutation resulted in a frame shift of *MECP2*, and premature termination of the 487 amino acid protein at the 154th codon, creating a large loss from the methyl-CpG binding domain to the C-terminal domain.

## Discussion

Approximately 80–90% of classic Rett syndrome patients harbor mutations in the coding region of *MECP2*. Furthermore, ~70% of Rett syndrome-causing *MECP2* mutations are C to T transitions at eight CpG dinucleotide mutation hotspots located within exons 3 and 4, and small deletions in the 3' end of exon 4 comprise ~10% of all mutated alleles (4, 15). Routine PCR-based DNA sequencing usually does not detect these deletions.

MLPA is useful for detecting large deletions in a single gene, but small deletions or polymorphisms that might affect the binding of the MLPA probe, resulting in a false positive, have occurred in Rett syndrome diagnosis (4). In addition, the mosaic could not be detected using MLPA. Therefore, it is necessary to identify MLPA results using other detection methods, such as real-time quantitative PCR and long-range PCR. Combined with the MLPA results, real-time quantitative PCR can narrow down the region of deletion. Primer sites in the dizygous regions immediately flanking the breakpoints were selected for long-range PCR amplification across the deletion junction, and DNA sequencing was performed to determine the accurate deletion region.

Long-range PCR products with heterozygous deletion mutations usually showed two DNA bands after agarose gel electrophoresis. The proband in our study revealed three bands. We first thought that the proband was a triple mosaicism for one normal *MECP2* gene allele and two different deletions alleles *in vivo*, resulting in the formation of three DNA bands after PCR *in vitro*. We initially suspected that a double strand DNA break occurred in *MECP2* during gene replication, and a variant of replication slippage occurred on both newly synthesized strands between the repeat motifs of microhomology, leading to the formation of the two different *MECP2* gene deletions *in vivo*, the similar mechanism has been reported in the *AR* gene (15). If this was truth, the *MECP2* deletions should be *de novo* mutations, occurring early in embryonic development of the proband. The probability of germline *MECP2* mosaicism in the parents could be ruled out, there seemed be no need to perform prenatal diagnosis for the proband's mother.

But by T-A cloning and comparing with the PCR product untreated and treated with heating and re-annealing, we found our previous assumption was wrong, the middle DNA band was proven to be heteroduplexes. Heteroduplexes are usually formed either during PCR cycling or by mixing of PCR product of mutant and wild-type DNAs *in vitro*, some large insertions or deletions may create stable heteroduplexes, and this stability is insensitive to variability in electrophoretic conditions (16). As far as we know, there is no evidence to show that a large number of heteroduplexes can exist *in vivo*. For the proband, the middle DNA band is just the heteroduplexes formed by PCR product *in vitro*, not *in vivo*. So the middle DNA band can't inherit from her mother. But *MECP2* deletion (bottom DNA band) of the proband really exists *in vivo*, and the deletion may not be *de novo*, it is probably inherited from the parent, who is germline mosaicism with *MECP2* deletion.

Although Rett syndrome is usually sporadic, it is commonly accepted that pregnant women who have a family history of Rett syndrome, should be offered an invasive prenatal diagnosis (17). Maternal and paternal *MECP2* germline mosaicism have been both reported in Rett syndrome families. Recently, Zhang et al. indicated that the cohort level of paternal germline *MECP2* mosaicism was 23.8% (5/21) by the detection of sperm (14). Meanwhile, maternal germline *MECP2* mosaicism was also verified in many Rett syndrome families, despite lack of the direct detection (2, 12, 13). In the case, we didn't find the *MECP2* deletion in the blood of the parents, and not found the *MECP2* mosaicism in the sperm of the proband's father (data not show), but the maternal germline mosaicism can't be ruled out. For the family, the recurrence risk of Rett syndrome still existed, it is necessary for the proband's mother to perform prenatal diagnosis.

In summary, we reported a novel *MECP2* heterozygous deletion in a Rett syndrome family. The proband was heterozygous for the deletion of 713-base pairs in exon 4 of *MECP2*, resulting in a frameshift and premature termination. To avoid recurrent risk, we performed prenatal diagnosis for her pregnant mother. Life is precious, which makes prenatal diagnosis a grave responsibility. It is necessary to obtain a clear molecular diagnosis, but each molecular diagnostic technique has its drawbacks. To avoid misdiagnosis, we performed prenatal diagnosis with multiple methods. In the case, we reported a novel heterozygous deletion in the *MECP2* gene with heteroduplexes of the PCR product *in vitro*, and provided a reference for the prenatal diagnosis of genetic diseases with complicated mutations, not limited to Rett syndrome.

## Data Availability Statement

The original contributions presented in the study are included in the article/Supplementary Material, further inquiries can be directed to the corresponding authors.

## Ethics Statement

The studies involving human participants were reviewed and approved by the Ethics Committee of Women's Hospital affiliated to the Zhejiang University School of Medicine. Written informed consent to participate in this study was provided by the participants' legal guardian/next of kin. Written informed consent was obtained from the individual(s), and minor(s)' legal guardian/next of kin, for the publication of any potentially identifiable images or data included in this article.

## Author Contributions

HZ, YS, YZ, JH, and MZ conducted experiments. YZ prepared the figures. YS and JH performed MLPA and PCR. MZ recruited samples. HZ and YZ wrote the manuscript. All authors read and approved the final manuscript.

## Funding

This study was supported by the National Natural Science Foundation of China (Grant No. 81801441) and the Technology Project of Zhejiang Provincial Health Commission (Grant No. 2021KY772).

## Conflict of Interest

The authors declare that the research was conducted in the absence of any commercial or financial relationships that could be construed as a potential conflict of interest.

## Publisher's Note

All claims expressed in this article are solely those of the authors and do not necessarily represent those of their affiliated organizations, or those of the publisher, the editors and the reviewers. Any product that may be evaluated in this article, or claim that may be made by its manufacturer, is not guaranteed or endorsed by the publisher.
