# Corrigendum: Case Report: Prenatal Diagnosis for a Rett Syndrome Family Caused by a Novel *MECP2* Deletion With Heteroduplexes of PCR Product

**DOI:** 10.3389/fped.2022.823860

**Published:** 2022-04-14

**Authors:** Honghong Zhang, Yixi Sun, Yuxia Zhu, Jiali Hong, Miaomiao Zheng

**Affiliations:** ^1^Department of Pediatrics, Hangzhou Children's Hospital, Hangzhou, China; ^2^Department of Reproductive Genetics, Women's Hospital, School of Medicine, Zhejiang University, Hangzhou, China; ^3^Key Laboratory of Reproductive Genetics, Ministry of Education, Zhejiang University, Hangzhou, China

**Keywords:** Rett syndrome, *MECP2*, prenatal diagnosis, mosaicism, heteroduplexes

There was an omission in the text of the original article, in **Case Presentation** section, paragraph six. We did not provide the transcript for the deletion of *MECP2*, our selected transcript was *MECP2* (NM_004992.4), and we have identified that the variation in our article was novel by searching in ClinVar, GnomAD, and the RettBase. A correction has been made from “In summary, we found a heterozygous deletion in c.441_1153del713 of *MECP2* in the proband.” to “In summary, we found a novel heterozygous deletion in c.441_1153del713 of *MECP2* (NM_004992.4) in the proband.”

In the original article, there was a mistake in Figure 1D. The base position number “1153” should be changed to “1154”. Additionally, there was also a mistake in Figure 1E. The images of Control group (PCR products untreated) were incorrect. We mistakenly replicated Heating and Reannealing group as Control group. The corrected Figure 1 appears below.

**Figure 1:**
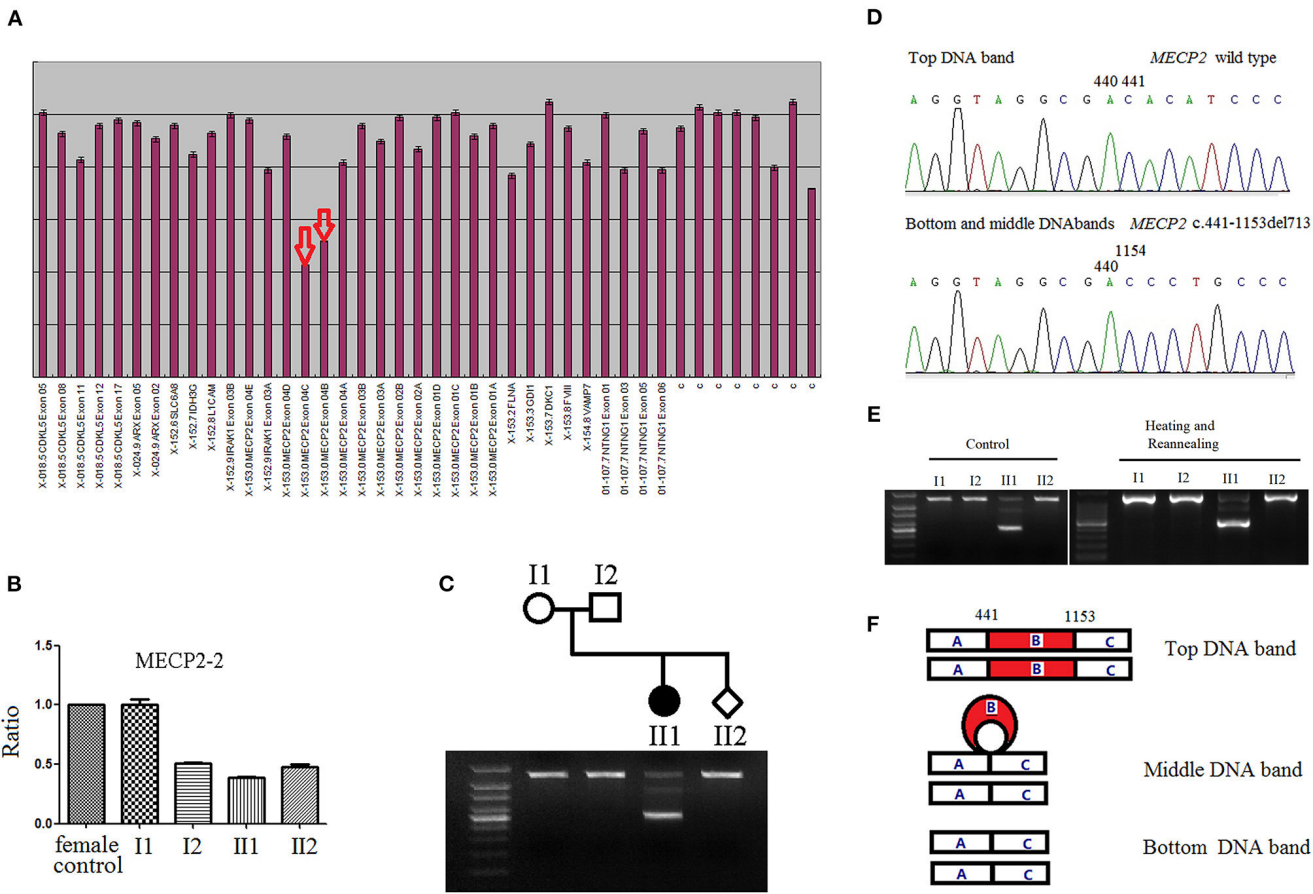
**(A)** Detection of MECP2 exon alterations via multiplex ligation-dependent probe amplification (MLPA). The arrows illustrate the presumed deletions, as the area of the peaks from the female proband DNA is approximately half that of the female controls. **(B)** The MECP2 amplicon with MECP2-real time quantitative PCR-2 primers to narrow down the deletion breakpoints and determine the results of MLPA. MECP2 relative amount of female proband, and male fetus were half that of female control. **(C)** The products of long-range PCR were analyzed with 1.5% agarose gel electrophoresis. The PCR product from the proband showed three DNA bands, whereas PCR product bands of the proband's parents and the fetus, were single. **(D)** Three DNA bands of long-range PCR from the proband were separately cut, T-A cloned, and finally sequenced after plasmid extraction. The sequence of top DNA band was wild type; both, bottom and middle DNA bands show 713-base pairs deletion in exon 4 (c.441_1153del713), and the single band of proband's parents and the fetus were of the wild type. **(E)** There was no difference between the PCR products untreated (left) and treated with heating and reannealing (right) in electrophoresis analysis. The treated PCR products were denatured at 95°C for 3 min, followed by gradual reannealing for 30 min with a temperature ramp of −1°C/min to optimize for the formation of heteroduplexes and homoduplexes. **(F)** Schematic diagram of three DNA bands after PCR. The top DNA band was homoduplex of wild type, the bottom DNA band was homoduplex of mutant type, the middle DNA band was heteroduplex of wild type and mutant type. Part B (red): 713-base pairs deletion in exon 4 (c.441_1153del713) of MECP2.

The authors apologize for these errors and state that these do not change the scientific conclusions of the article in any way. The original article has been updated.

## Publisher's Note

All claims expressed in this article are solely those of the authors and do not necessarily represent those of their affiliated organizations, or those of the publisher, the editors and the reviewers. Any product that may be evaluated in this article, or claim that may be made by its manufacturer, is not guaranteed or endorsed by the publisher.

