# Supplementary figures and images for: Case Report: Prenatal Diagnosis for a Rett Syndrome Family Caused by a Novel MECP2 Deletion With Heteroduplexes of PCR Product
Source: Front Pediatr. 2021 Oct 27;9:748641. doi: 10.3389/fped.2021.748641 (PMC8578848; doi:10.3389/fped.2021.748641)

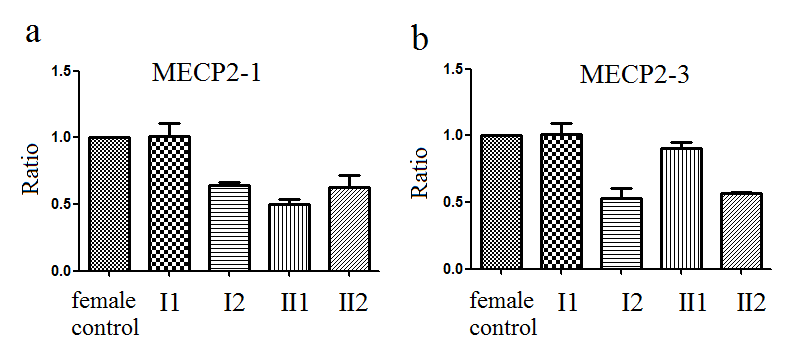

Supplement: Supplementary file 1 [file Image_1.TIF]
