# Supplementary material for: Case Report: Prenatal Diagnosis for a Rett Syndrome Family Caused by a Novel MECP2 Deletion With Heteroduplexes of PCR Product
Source: Front Pediatr. 2021 Oct 27;9:748641. doi: 10.3389/fped.2021.748641 (PMC8578848; doi:10.3389/fped.2021.748641)
Supplement: Supplementary file 2 [file Data_Sheet_1.docx]

1. The primers lists

| Primer | DNA sequences |
| --- | --- |
| β-globin- real time PCR-F | ACACAACTGTGTTCACTAGC |
| β-globin- real time PCR-R | CAACTTCATCCACGTTCACC |
| MECP2 -real time PCR-1-F | TAAAGTGGAGTTGATTGCGTAC |
| MECP2- real time PCR-1-R | TTGGGCTTCTTAGGTGGTT |
| MECP2- real time PCR-2-F | GGAGTCTTCTATCCGATCTGTG |
| MECP2- real time PCR-2-R | CCTTTCCCGCTCTTCTCA |
| MECP2 -real time PCR-3-F | GACATTGTTTCATCCTCCAT |
| MECP2- real time PCR-3-R | TTTGTCAGAGCCCTACCC |
| MECP2- long-range PCR-F | GCAGGCAGACGAGTGAGT |
| MECP2- long-range PCR-R | CTTGGCATGGAGGATGAA |

2. Genomic sequence near the deletion of MECP2 exon4.Exon 4 of MECP2 is in upper case. Intron is in lower case.

aattccatgagaagggtgagcaaaggattatcttgttgaaactgattcctggagagactgagcaccgtacctgagttcaaacttgggaatgttctagatggtgactcaggcccaggcaccaaccagcagaatgggcctcagcctgacaacccttctgtaccaggcctgactctttggttgctgaactttggagaggcctgggggggtcagcggcaggcagacgagtgagtggctttggtgacaggtcctcaggggcagccaggcagtgtgactctcgttcaatagtaacgtttgtcagagcgttgtcaccaccatccgctctgccctatctctgacattgctatggagagcctctaattgttccttgtgtctttctgtttgtccccacagTCCCCAGGGAAAAGCCTTTCGCTCTAAAGTGGAGTTGATTGCGTACTTCGAAAAGGTAGGCGACACATCCCTGGACCCTAATGATTTTGACTTCACGGTAACTGGGAGAGGGAGCCCCTCCCGGCGAGAGCAGAAACCACCTAAGAAGCCCAAATCTCCCAAAGCTCCAGGAACTGGCAGAGGCCGGGGACGCCCCAAAGGGAGCGGCACCACGAGACCCAAGGCGGCCACGTCAGAGGGTGTGCAGGTGAAAAGGGTCCTGGAGAAAAGTCCTGGGAAGC**TCCTTGTCAAGATGCCTTTTCAAA**CTTCGCCAGGGGGCAAGGCTGAGGGGGGTGGGGCCACCACATCCACCCAGGTCATGGTGATCAAACGCCCCGGCAGGAAGCGAAAAGCTGAGGCCGACCCTCAGGCCATTCCCAAGAAACGGGGCCGAAAGCCGGGGAGTGTGGTGGCAGCCGCTGCCGCCGAGGCCAAAAAGAAAGCCGTGAAGGAGTCTTCTATCCGATCTGTGCAGGAGACCGTACTCCCCATCAAGAAGCGCAAGACCCGGGAGACGGTCAGCATCGAGGTCAAGGAAGTGGTGAAGCCCCTGCTGGTGTCCACCCTCGGTGAGAAGAGCGGGAAAGGA**CTGAAGACCTGTAAGAGCCCTGGG**CGGAAAAGCAAGGAGAGCAGCCCCAAGGGGCGCAGCAGCAGCGCCTCCTCACCCCCCAAGAAGGAGCACCACCACCATCACCACCACTCAGAGTCCCCAAAGGCCCCCGTGCCACTGCTCCCACCCCTGCCCCCACCTCCACCTGAGCCCGAGAGCTCCGAGGACCCCACCAGCCCCCCTGAGCCCCAGGACTTGAGCAGCAGCGTCTGCAAAGAGGAGAAGATGCCCAGAGGAGGCTCACTGGAGAGCGACGGCTGCCCCAAGGAGCCAGCTAAGACTCAGCCCGCGGTTGCCACCGCCGCCACGGCCGCAGAAAAGTACAAACACCGAGGGGAGGGAGAGCGCAAAGACATTGTTTCATCCTCCATGCCAAGGCCAAACAGAGAGGAGCCTGTGGACAGCCGGACGCCCGTGACCGAGAGAGTTAGCTGActttacacggagcggattgcaaagcaaaccaacaagaataaaggcagctgttgtctcttctccttatgggtagggctctgacaaagcttcccgattaactgaaataaaaaatattttt

(1)

TCCTTGTCAAGATGCCTTTTCAAA probe 10842-L12494 24 nt adjacent to ligation site

CTGAAGACCTGTAAGAGCCCTGGG probe 01347-L12498 24 nt adjacent to ligation site

hemizygous copy number of the proband for MLPA probes (MRC-Holland, kit P015C)

(2)----- ----- ----- MECP2-real time PCR-1/2/3-foword primer and the reverse complement of its reverse primer

(3) gcaggcagacgagtgagt MECP2- long-range PCR foword primer

TTCATCCTCCATGCCAAG reverse complement of MECP2-long-range PCR reverse primer

(4) ----- the heterozygous deletion regions of the proband

3. Supplement Fig. 1 Legends

(a) The amplicon with *MECP2*-real time PCR-1 primers showed that the relative dosage of the proband was half that of normal female samples, implying that the primers were in the region of heterozygous deletion. Thus, the designing of long-range PCR forward primer in the upper region should be attempted.

(b) The amplicon with *MECP2*-real time PCR-3 primers showed that the relative dosage of the proband was 1, indicating that the region was not deleted, and the long-range PCR reverse primer could be in the downstream region.

The two figures show that the parents and the fetus (male) were normal, without deletions. The dosage ratio of each involved *MECP2* exon was calculated using the ΔΔCt method for each test sample, and an autosomal gene β-globin (HBB) was selected as a reference gene. The 2−(ΔΔCt ± SD) value of ~0.5 is expected for female heterozygotes with deletions or normal males.
